# Early prophylactic anticoagulation with heparin alleviates mortality in critically ill patients with sepsis: a retrospective analysis from the MIMIC-IV database

**DOI:** 10.1093/burnst/tkac029

**Published:** 2022-09-23

**Authors:** Zhi-ye Zou, Jia-jia Huang, Ying-yi Luan, Zhen-jia Yang, Zhi-peng Zhou, Jing-jing Zhang, Yong-ming Yao, Ming Wu

**Affiliations:** Department of Critical Care Medicine and Hospital Infection Prevention and Control, Shenzhen Second People’s Hospital & First Affiliated Hospital of Shenzhen University, Shenzhen 518035, China; Department of Critical Care Medicine and Hospital Infection Prevention and Control, Shenzhen Second People’s Hospital & First Affiliated Hospital of Shenzhen University, Shenzhen 518035, China; Postgraduate Education, Shantou University Medical College, Shantou 515041, China; Department of Central Laboratory, Beijing Obstetrics and Gynecology Hospital, Capital Medical University, Beijing 100026, China; Department of Critical Care Medicine and Hospital Infection Prevention and Control, Shenzhen Second People’s Hospital & First Affiliated Hospital of Shenzhen University, Shenzhen 518035, China; Postgraduate Education, Shantou University Medical College, Shantou 515041, China; Department of Critical Care Medicine and Hospital Infection Prevention and Control, Shenzhen Second People’s Hospital & First Affiliated Hospital of Shenzhen University, Shenzhen 518035, China; Department of Critical Care Medicine and Hospital Infection Prevention and Control, Shenzhen Second People’s Hospital & First Affiliated Hospital of Shenzhen University, Shenzhen 518035, China; Postgraduate Education, Shantou University Medical College, Shantou 515041, China; Trauma Research Center, Medical Innovation Research Department and Fourth Medical Center of the Chinese PLA General Hospital, Beijing 100048, China; Department of Critical Care Medicine and Hospital Infection Prevention and Control, Shenzhen Second People’s Hospital & First Affiliated Hospital of Shenzhen University, Shenzhen 518035, China; Postgraduate Education, Shantou University Medical College, Shantou 515041, China; Guangxi University of Chinese Medicine, Nanning 530200, China

**Keywords:** Early prophylactic anticoagulation, Heparin, Mortality, Sepsis, Critically ill patients

## Abstract

**Background:**

Minimal data exist on anticoagulation use and timing and the dose of heparin in patients with sepsis, and whether heparin use improves sepsis survival remains largely unclear. This study was performed to assess whether heparin administration would provide a survival advantage in critically ill patients with sepsis.

**Methods:**

A retrospective cohort study of patients with sepsis in the Medical Information Mart for Intensive Care (MIMIC)-IV database was conducted. Cox proportional hazards model and propensity score matching (PSM) were used to evaluate the outcomes of prophylactic anticoagulation with heparin administered by subcutaneous injection within 48 h of intensive care unit (ICU) admission. The primary outcome was in-hospital mortality. Secondary outcomes included 60-day mortality, length of ICU stay, length of hospital stay and incidence of acute kidney injury (AKI) on day 7. E-Value analysis were used for unmeasured confounding.

**Results:**

A total of 6646 adult septic patients were included and divided into an early prophylactic heparin group (*n* = 3211) and a nonheparin group (*n* = 3435). In-hospital mortality in the heparin therapy group was significantly lower than that in the nonheparin group (prematched 14.7 *vs* 20.0%, hazard ratio (HR) 0.77, 95% confidence interval (CI) [0.68–0.87], *p* < 0.001, and postmatched 14.9 *vs* 18.3%, HR 0.78, 95% CI [0.68–0.89], *p* < 0.001). Secondary endpoints, including 60-day mortality and length of ICU stay, differed between the heparin and nonheparin groups (*p* < 0.01). Early prophylactic heparin administration was associated with in-hospital mortality among septic patients in different adjusted covariates (HR 0.71–0.78, *p* < 0.001), and only administration of five doses of heparin was associated with decreased in-hospital mortality after PSM (HR 0.70, 95% CI 0.56–0.87, *p* < 0.001). Subgroup analysis showed that heparin use was significantly associated with reduced in-hospital mortality in patients with sepsis-induced coagulopathy, septic shock, sequential organ failure assessment score ≥ 10, AKI, mechanical ventilation, gram-positive bacterial infection and gram-negative bacterial infection, with HRs of 0.74, 0.70, 0.58, 0.74, 0.73, 0.64 and 0.72, respectively (*p* <0.001). E-Value analysis suggested robustness to unmeasured confounding.

**Conclusions:**

This study found an association between early administration prophylactic heparin provided to patients with sepsis and reduced risk-adjusted mortality. A prospective randomized-controlled study should be designed to further assess the relevant findings.

Highlights

- Early administration prophylactic heparin could reduce in-hospital mortality and 60-day mortality in patients with sepsis.
- Administration of five doses of heparin was associated with decreased in-hospital mortality after PSM.
- Heparin use was significantly associated with reduced in-hospital mortality in patients with sepsis-induced coagulopathy, septic shock, SOFA score ≥ 10, acute kidney injury and mechanical ventilation, with HRs of 0.74, 0.70, 0.58, 0.74 and 0.73 respectively (*p* <0.001).

## Background

Infection, inflammation and blood coagulation are inextricable in patients with sepsis [1]. Severe infection activates the coagulation cascade among patients with sepsis [2]. In addition, coagulopathy is a powerful generator or amplifier of inflammatory responses [3]. Both coagulopathy and inflammatory cytokines are closely related to the prognosis of sepsis [4]. In the early stage of sepsis, immune thrombosis facilitates bacterial elimination, while disordered thrombi might augment multiple organ dysfunction [5]. Therefore, the control of inflammation and coagulation is crucial for the management of sepsis [6].

As an anticoagulation agent, heparin has been widely used in the clinic for several decades. In a prospective, randomized, double-blind study, unfractionated heparin did not affect 28-day mortality in septic patients, did not reduce organ failure scores and was associated with more extended hospital stays [7]. However, Protein C Worldwide Evaluation of Severe Sepsis (PROWESS, *n* = 1690) demonstrated that heparin therapy was associated with 28-day survival (odds ratio 0.6) compared with no heparin prescription [7,8]. Three systematic reviews also showed that treatment with a low dose of heparin was associated with significantly reduced 28-day mortality in patients with sepsis [9–11]. Thus, the efficacy of heparin in septic patients followed by various complications remains unclear. Although animal studies have demonstrated that heparin can combine with lipopolysaccharide to reduce the mortality resulting from gram-negative bacterial infection, this has not been proven clinically.

The timing and dose of heparin and the type of pathogen causing the infection affect the efficacy of heparin in the treatment of sepsis. To evaluate the effectiveness of prophylactic heparin for patients with sepsis, we used the Medical Information Mart for Intensive Care (MIMIC) IV database to assess whether prophylactic heparin administered to septic patients within 48 h after intensive care unit admission is associated with lower in-hospital mortality and to further estimate the differential effect of heparin on gram-positive or gram-negative bacterial infections.

## Methods

### Data source and study design

We performed a retrospective cohort study using data from MIMIC IV (MIMIC-IV version 1.0, [12]), which includes two in-hospital database systems: a custom hospital-wide electronic health record (EHR) and an intensive care unit (ICU)-specific clinical information system from 2008 to 2019. Individuals who complete the Collaborative Institutional Training Initiative examination (Certification number 35951237 for author ZYZ) can access the database. De-identification was performed to ensure patient confidentiality. This study was approved by the Research Ethics Committee of Shenzhen Second People’s Hospital (20210831001).

### Participants

All patients with sepsis were included from the MIMIC-IV database, for a total of 25467 patients. The inclusion criteria were anti-infective treatment within 6 h and a sequential organ failure assessment (SOFA) score ≥ 2 within 24 h after ICU admission. The exclusion criteria were as follows: age < 18 years; emergency surgical treatment; use of heparin for dialysis or treatment, rather than for prophylactic use; use of enoxaparin or warfarin; exposure to heparin 48 h after ICU admission; and ICU stay <48 h. For patients who were admitted to the ICU more than once, we included only the first ICU admission data from the first hospital stay.

### Research procedures and definitions

The data were extracted from MIMIC-IV using Structured Query Language with Navicat Premium (version 12.0.28) and consisted of age, sex, ethnicity, insurance, weight, history of disease, Charlson comorbidity index (CCI), SOFA score, simplified acute physiology score II (SAPS II), renal replacement therapy (RRT), mechanical ventilation use, sepsis-induced coagulation (SIC), septic shock, positive fluid balance, acute kidney injury (AKI), gram-positive bacterial infection and gram-negative bacterial infection. Information on the use of anticoagulant drugs comprised drug name, dose, route, start time and end time. Anticoagulant drugs included heparin, enoxaparin and warfarin. Other drugs, such as low molecular weight heparin, dalteparin, tinzaparin and thrombomodulin, were not found in MIMIC-IV. The early prophylactic use of heparin (within 48 h of ICU admission) was documented by the medical order system and excluded heparin for other purposes, such as dialysis or treatment. Only the subcutaneous injection route was used, and one dose was 5000 units. We also excluded patients who used enoxaparin, warfarin and other anticoagulants during hospitalization.

In this study, sepsis was defined as patients with a documented or suspected infection plus SOFA scores with an acute increase of ≥2 [13]. Septic shock is defined as sepsis associated with hypotension and perfusion abnormalities despite the provision of adequate fluid resuscitation [13]. We used the methods of other previous studies to analyze this database (sepsis and septic shock) and analyzed the extracted patient data [14]. Fluid positivity means that the amount of crystals plus colloids is greater than the urine output within 24 h after ICU admission [15]. Gram-positive bacteria and gram-negative bacteria refer to the types of microorganisms cultivated within 48 h after ICU admission.

### Outcomes and measures

The primary outcome was in-hospital mortality. Secondary outcomes included 60-day mortality, length of ICU stay, length of hospital stay and incidence of AKI on day 7.

### Statistical analysis

Continuous variables in the current study are expressed as the mean ± standard deviation (SD), and the differences between groups were identified with a t-test. Categorical variables are expressed as numbers (percentage), and comparisons between groups were made using the chi-square test or Fisher’s exact test as appropriate. Risk factors were assessed for an association with in-hospital mortality with a Cox proportional hazards model. Propensity score matching (PSM) was conducted to balance the baseline characteristics between the nonheparin and early heparin use groups. Thus, we used a logistic regression model to calculate the propensity score for each patient and performed 1:1 matching for the two groups. After PSM, standardized mean differences (SMDs) and *p* values were used to evaluate the balance of characteristics between the two groups. A variable could be considered imbalanced between the groups when its SMD was >0.1 [16].

We calculated the in-hospital mortality absolute risk reduction on the basis of heparin prescriptions. We also explored the potential for unmeasured confounding between early prophylactic heparin prescriptions and mortality by calculating E-values [17]. The E-value quantifies the required magnitude of an unmeasured confounder that could negate the observed association between heparin and mortality.

An extended Cox model approach was used for adjustment of the following covariates: SOFA, SIC, septic shock, AKI, mechanical ventilation, gram-positive bacterial infection and gram-negative bacterial infection. All of the covariates were important factors affecting the decision to use heparin in clinical practice. The effect of heparin dose on in-hospital mortality was also evaluated with a Cox regression model before and after PSM. Survival analysis for patients with and without heparin was performed using Kaplan–Meier analysis and log-rank tests before and after PSM.

Stratification analysis was conducted to explore whether heparin administration and in-hospital mortality differed across the various subgroups classified by SOFA, SIC, septic shock, AKI, mechanical ventilation, gram-positive bacterial infection and gram-negative bacterial infection. Subgroup analysis also used a Cox model adjusted for all variables in the patient baseline information. Multiple imputations were used for missing values under the assumption of missing at random [18]. Two-tailed *p* values < 0.05 were considered to indicate statistical significance. All statistical analyses were performed using Stata 15.1 (StataCorp, College Station, TX, USA) and R 4.0.1 software for windows.

**Figure 1.**
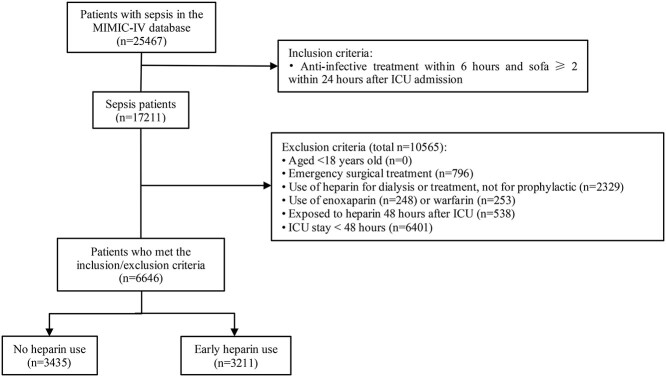
Diagram of patient eligibility and inclusion. *ICU* intensive care unit, *MIMIC* Medical Information Mart for Intensive Care

**Table 1:** Baseline characteristics of patients with sepsis before and after propensity score matching

**Patient characteristic**		**Propensity score matching**						
		**Before**				**After**		
	**All patients (*n* = 6646)**	**No heparin (*n* = 3435)**	**Early heparin (*n* = 3211)**	***P* value**		**No heparin (*n* = 2708)**	**Early heparin (*n* = 2708)**	***P* value**
Age (years), mean (SD)	66.29 (16.52)	66.21 (16.1)	66.38 (16.9)	0.68		66.64 (16.5)	66.42(16.9)	0.63
Male, *n* (%)	3658 (55.0)	1974 (57.5)	1684 (52.4)	<0.001		1483 (54.8)	1481 (54.7)	0.96
White, *n* (%)	4381 (65.9)	2278 (66.3)	2103 (65.5)	0.48		1795 (66.3)	1783 (65.8)	0.73
Insurance, Medicare, *n* (%)	3371 (50.7)	1647 (47.9)	1724 (53.7)	<0.001		1411 (52.1)	1406 (51.9)	0.89
Weight (kg), mean (SD)	81.06 (24.39)	80.9 (23.6)	81.3 (25.4)	0.54		80.99 (24.1)	80.96 (24.4)	0.97
History of disease, *n* (%)								
Hypertension	4459 (67.1)	2366 (68.9)	2093 (65.2)	0.001		1794 (66.2)	1802 (66.5)	0.82
Diabetes	2215 (33.3)	1112 (32.4)	1103 (34.4)	0.087		909 (33.6)	911 (33.6)	0.95
Chronic pulmonary disease	1965 (29.6)	936 (27.2)	1029 (32.0)	<0.001		800 (29.5)	805 (29.7)	0.88
CKD	1686 (25.4)	913 (26.6)	773 (24.1)	0.019		689 (25.4)	684 (25.3)	0.88
Scoring system, mean (SD)								
CCI	6.09 (2.99)	6.27 (2.99)	5.89 (2.97)	<0.001		6.05 (2.9)	6.03 (3.00)	0.80
Maximum SOFA score on 1st day	7.54 (3.83)	7.81 (4.03)	7.26 (3.57)	<0.001		7.47 (3.9)	7.48 (3.61)	0.91
Maximum SAPS II on 1st day	42.10 (13.95)	42.27 (13.9)	41.92 (14.0)	0.30		42.33 (14.0)	42.21(14.0)	0.74
RRT on day 1, *n* (%)	444 (6.7)	166 (4.8)	112 (3.5)	0.006		114 (4.2)	107 (4.0)	0.63
Mechanical ventilation, *n* (%)	3363 (50.6)	1717 (50.0)	1646 (51.3)	0.30		1340 (49.5)	1365 (50.4)	0.50
SIC, *n* (%)	5560 (83.7)	3074 (89.5)	2486 (77.4)	<0.001		2347 (86.7)	2342 (86.5)	0.84
Septic shock, *n* (%)	2589 (39.0)	1368 (39.8)	1221 (38.0)	0.13		1050 (38.8)	1060 (39.1)	0.78
Positive fluid balance, *n* (%)	4003 (60.2)	2000 (58.2)	2003 (62.4)	<0.001		1673 (61.8)	1661 (61.3)	0.74
Gram-positive bacteria, *n* (%)	1313 (19.8)	603 (17.6)	710 (22.1)	<0.001		547 (20.2)	537 (19.8)	0.73
Gram-negative bacteria, *n* (%)	1020 (15.3)	475 (13.8)	545 (17.0)	<0.001		415 (15.3)	427 (15.8)	0.65

*CKD* chronic kidney disease, *CCI* Charlson comorbidity index, *SOFA* sequential organ failure assessment, *SAPS II* simplified acute physiology score II, *RRT* renal replacement therapy, *SIC* sepsis-induced coagulopathy

## Results

### Patient characteristics

The MIMIC-IV database included 25,467 patients with sepsis. After exclusion of patients who met the exclusion criteria, 6646 eligible patients were enrolled. A total of 3211 patients were administered heparin within the first 48 h after ICU admission and 3435 patients did not receive heparin treatment (Figure 1). Among the 6646 patients, the mean (SD) age was 66.29 (16.52) years, 3658 (55.0%) were male, 4381 (65.9%) were White individuals, the mean (SD) body weight was 81.06 (24.39) kg, 1313 (19.8%) had gram-positive bacterial infection and 1020 (15.3%) had gram-negative bacterial infection. Approximately half of all patients (3211 [48.3%]) were treated with early prophylactic heparin (Table 1).

There were no significant differences between the two groups in age, ethnicity, weight, history of diabetes, SAPS II score, mechanical ventilation use or septic shock (*p* > 0.05). The proportion of men; percentages of patients with a history of hypertension, chronic kidney disease (CKD), RRT and SIC; CCI scores; and SOFA scores in the heparin group were lower than those in the nonheparin group (*p* < 0.001). However, chronic lung disease, positive fluid balance and positivity rates of gram-positive and gram-negative bacteria were higher in the early heparin group than in the nonheparin group (*p* < 0.001) (Table 1). After PSM, the SMDs were all <0.1, indicating that the baseline variables in the two groups had similar distributions (Table 1, Figure S1, see online supplementary material).

**Table 2:** Association between early heparin use and outcomes in septic patients

	**Propensity score matching cohort (*n* = 5416)**							**All cohort (*n* = 6646)**			
**Outcome**	**No heparin *n* = 2708**	**Early heparin *n* = 2708**	**ARR** ^**a**^ **(95% CI)**	***P* value**	**Matched HR (95% CI)** ^**b**^	***P* value**		**No heparin *n* = 3435**	**Early heparin *n* = 3211**	**Adjusted HR (95%CI)** ^**c**^	***P* value**
Primary											
In-hospital mortality, *n* (%)	496 (18.3)	403 (14.9)	3.43(1.45,4.41)	<0.001	0.78 (0.68, 0.89)	<0.001		686 (20.0)	471 (14.7)	0.77(0.68, 0.87)	<0.001
Secondary											
60-day mortality, *n* (%)	534 (19.7)	448 (16.5)	3.18(1.13, 5.23)	0.002	0.81 (0.72, 0.92)	0.001		731 (21.3)	515 (16.0)	0.80(0.71, 0.89)	<0.001
Length of ICU stay, mean (SD)	4.97 (4.6)	5.94 (5.22)	NA	<0.001	NA	NA		5.09 (4.72)	5.95 (5.29)	NA	<0.001
Length of hospital stay, mean (SD)	12.41 (12.6)	12.75(12.6)	NA	0.32	NA	NA		12.71 (13.2)	12.68 (12.4)	NA	0.91
AKI, *n* (%)	1223 (45.2)	1223 (45.2)	NA	1.00	NA	NA		1607 (46.8)	1421 (44.3)	NA	0.039
AKI stage 3, *n* (%)	280 (10.3)	293 (10.8)	NA	0.57	NA	NA		383 (11.1)	323 (10.1)	NA	0.15

*ARR* Absolute risk reduction, *HR* hazard ratio, *NA* not applicable, *AKI* acute kidney injury

^a^ARR excluded for length of ICU stay, length of hospital stay and incidence of AKI on day 7.

^b^Results of univariable analysis of propensity score matched cohort.

^c^Adjusted results obtained from multivariable Cox proportional hazards regression model that included the full cohort

### Outcomes

**Figure 2.**
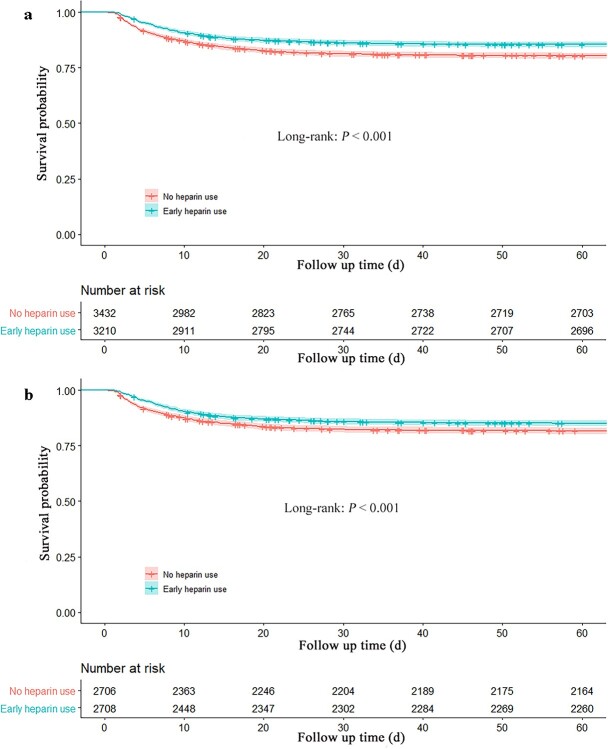
Kaplan–Meier survival curve of the two groups before (**a**) and after (**b**) propensity score matching

The prematched crude hospital mortality rate was significantly lower in patients with early heparin use than in those without heparin use (14.7 *vs* 20.0%, hazard ratio (HR) 0.77, 95% confidence interval (CI) [0.68–0.87] *p* < 0.001). After PSM, similar to the results in the prematched model, heparin was associated with reduced in-hospital mortality (14.9 *vs* 18.3%, absolute risk reduction 3.43 [95% CI, 1.45–4.41], HR 0.78, 95% CI [0.68–0.89], *p* < 0.001) (Table 2). The 60-day mortality rate in the early heparin group was lower than that in the nonheparin group (prematched 16.0 *vs* 21.3%, HR 0.80, 95% CI [0.71–0.89], postmatched 16.5 *vs* 19.7%, HR 0.81, 95% CI [0.72–0.92], *p* < 0.01), and the Kaplan–Meier curves showed a significant difference between early heparin use and nonheparin use before and after PSM (*p* < 0.001) (Table 2, Figure 2).

The length of ICU stay in the early heparin group was longer than that of the nonheparin group (prematched 5.29 *vs* 4.72 days, postmatched 5.33 *vs* 4.63 days, *p* < 0.001). There was no significant difference in the length of hospital stay between the two groups (*p* > 0.05). The incidence of AKI in the early heparin group was lower than that in the nonheparin group before matching (Table 2).

In the extended multivariable Cox proportional hazards models, HR of early heparin use was consistently significant in five models after adjustment for covariates (HR range 0.71–0.78, all *p* < 0.001). In-hospital mortality in the heparin group tended to decrease with the increased use of heparin doses before PSM (*p* for trend <0.001), and only administration of ≥5 doses/48 h was associated with a reduced risk of in-hospital mortality after PSM (HR 0.700; 95% CI 0.562–0.872; *p* = 0.001) (Table 3).

**Table 3:** Efficacy of early heparin use in in-hospital mortality and dose used

**Variables**	**Hazard ratio**	**95% Confidence interval**	***P* value**
Model 1	0.708	0.630–0.796	<0.001
Model 2	0.714	0.635–0.803	<0.001
Model 3	0.779	0.691–0.878	<0.001
Model 4	0.787	0.698–0.889	<0.001
No heparin use	1.000 (reference)		
Early heparin use (dose^a^/48 h)			
**Prematched cohort**			
1–2	0.752	0.634–0.892	<0.001
3–4	0.723	0.616–0.848	<0.001
≥5	0.621	0.509–0.757	<0.001
*P* value trend^b^	<0.001		
**Postmatched cohort**			
1–2	0.834	0.692–1.004	0.055
3–4	0.843	0.706–1.005	0.057
≥5	0.700	0.562–0.872	0.001
*P* value trend^b^	<0.001		

Adjusted covariates: Model 1 = early heparin use. Model 2 = Model 1 + age, sex, ethnicity, insurance, weight + (history of disease including hypertension, diabetes, chronic pulmonary disease and chronic kidney disease). Model 3 = Model 2 + (scoring system including Charlson comorbidity index, sequential organ failure assessment and simplified acute physiology score II). Model 4 = Model 3 + sepsis-induced coagulation, septic shock and positive fluid balance.

^a^1 Dose of heparin = 5000 units of heparin, prophylactic subcutaneous injection. ^b^*P* value trend, from a one degree-of-freedom trend test

**Figure 3.**
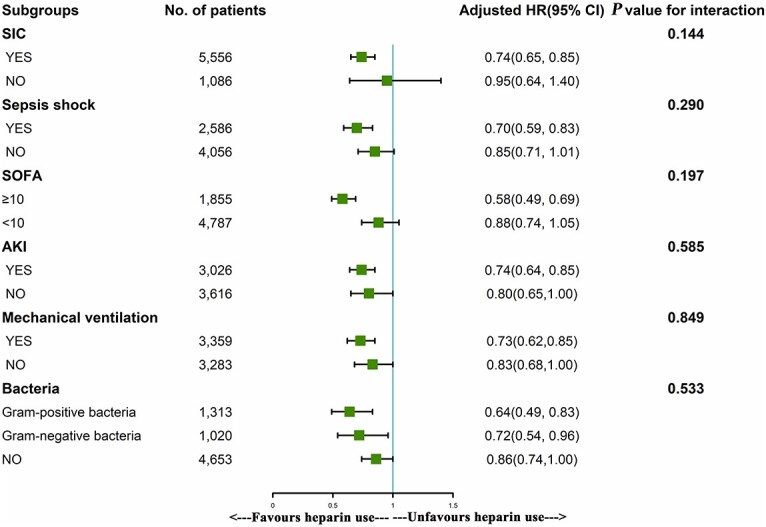
The association between early heparin use and in-hospital mortality in subgroups. *AKI* acute kidney injury, *HR* hazard ratio, *SIC* sepsis-induced coagulation, *SOFA* sequential organ failure assessment

Subgroup analysis showed that the use of heparin was significantly associated with reduced in-hospital mortality in patients who had SIC, septic shock, a SOFA score ≥ 10, AKI, mechanical ventilation use, a gram-positive bacterial infection and a gram-negative bacterial infection, with HRs of 0.74, 0.70, 0.58, 0.74, 0.73, 0.64 and 0.72, respectively (*p* < 0.001) (Figure 3).

### Sensitivity analysis

Significant known and measured risk factors for in-hospital mortality after PSM within the multivariable Cox-proportional hazard model included age (HR, 1.013 [95% CI, 1.009–1.017]), Insurance Medicare (HR, 1.177 [95% CI, 1.032–1.343]), CKD (HR, 1.229 [95% CI 1.063–1.420]), CCI (HR, 1.135 [95% CI 1.111–1.159]), maximum SOFA score on day 1 (HR, 1.189 [95% CI 1.171–1.207]), maximum SAPS II on day 1 (HR, 1.047 [95% CI 1.043–1.051]), mechanical ventilation use (HR, 1.491 [95% CI 1.305–1.703]), SIC (HR, 1.550 [95% CI 1.238–1.940]), septic shock (HR, 1.786 [95% CI 1.566–2.036]), positive fluid balance (HR, 1.427 [95% CI 1.239–1.644]), gram-positive bacteria (HR, 1.312 [95%CI 1.126–1.528]) and gram-negative bacteria (HR, 1.254 [95%CI 1.060–1.485]) (Table 4).

**Table 4:** Risk factors for in-hospital mortality in septic patients

**Variable**	**HR**	**95% CI**	***P* value**
Age	1.013	1.009–1.017	<0.001
Male	0.933	0.818–1.064	0.301
White	0.881	0.769–1.009	0.068
Insurance, Medicare	1.177	1.032–1.343	0.015
Weight (kg)	0.997	0.994–0.999	0.045
Comorbidities			
Hypertension	1.038	0.903–1.194	0.600
Diabetes	0.889	0.772–1.025	0.104
Chronic pulmonary disease	1.051	0.912–1.212	0.494
CKD	1.229	1.063–1.420	0.005
CCI	1.135	1.111–1.159	<0.001
Maximum SOFA score on day 1	1.189	1.171–1.207	<0.001
Maximum SAPS II on day 1	1.047	1.043–1.051	<0.001
Renal replacement therapy on day 1	1.188	0.8655–1.632	0.286
Mechanical ventilation	1.491	1.305–1.703	<0.001
SIC	1.550	1.238–1.940	<0.001
Septic shock	1.786	1.566–2.036	<0.001
Positive fluid balance	1.427	1.239–1.644	<0.001
Gram-positive bacteria	1.312	1.126–1.528	<0.001
Gram-negative bacteria	1.254	1.060–1.485	0.008

A Cox proportional hazards model showed that age, chronic kidney disease (CKD), Charlson comorbidity index (CCI) score, sequential organ failure assessment (SOFA) score, simplified acute physiology score II (SAPS II), mechanical ventilation, sepsis-induced coagulation (SIC), septic shock, positive fluid balance, gram-positive bacterial infection and gram-negative bacterial infection were independent risk factors for in-hospital mortality after propensity score matching, *CI* confidence interval

We generated an E-value to assess the sensitivity to unmeasured confounding. (https://www.evalue-calculator.com/evalue/). COX analysis found septic shock (HR1.786) was the highest risk factor, which is < 1.88, that means there were no undetermined risk factors affect the results, with an HR > 1.88 (upper limit 3.39), meaning that residual confounding could explain the observed association if there exists an unmeasured covariate having a relative risk association >1.88 with both in-hospital mortality and early prophylactic heparin prescriptions. Therefore, it is unlikely that an unmeasured or unknown confounder would have a substantially greater effect on in-hospital mortality (relative risk > 1.88) than these known risk factors.

## Discussion

Increasing evidence indicates an extensive interaction between inflammation and coagulation that may play a vital role in the pathophysiology of sepsis [19].Coagulation dysfunction and inflammatory disorders increase the morbidity and mortality of patients with sepsis. Nevertheless, whether regulating coagulation function can improve the prognosis of septic patients remains controversial.

In an open-label, adaptive, multiplatform, randomized clinical trial, therapeutic-dose anticoagulation with heparin did not result in a greater probability of survival to hospital discharge or a greater number of days free of respiratory organ support in critically ill patients with sepsis-associated COVID-19 [20]. In the Phase III Tifacogin in Multicenter International Sepsis Trial (OPTIMIST, *n* = 1754), a subgroup study demonstrated that heparin therapy did not improve 28-day survival (odds ratio 1.07 [0.83–1.38], *p* = 0.62 [21]. Some studies have even pointed out that heparin cannot influence the cascade of inflammation, thrombosis and organ injury in patients with advanced disease [22,23]. Moreover, heparin possesses other properties in addition to anticoagulation, including anti-inflammatory actions, anti-complement activity and modulation of various proteases [24,25]. Several systematic reviews have shown a favorable association between heparin use and survival from sepsis [9–11]. In another open-label, adaptive, multiplatform, controlled trial, therapeutic-dose anticoagulation with heparin increased the probability of survival to hospital discharge in noncritically ill patients [26]. A nationwide cohort also showed that early prophylactic anticoagulation among patients with COVID-19 was associated with a decreased risk of 30-day mortality [27]. Herein, our data suggested that early prophylactic heparin use was significantly associated with decreased in-hospital mortality in patients with sepsis. This result was robust in the PSM analysis, which reduced the confounding bias. What factors account for the different results? Timing, dose, type of anticoagulant agent, microorganism species and host factors might affect the outcomes.

The coagulation system and innate immune system work closely together [23]. Inflammation and coagulation are inextricably linked, and this interaction contributes to the pathophysiology of sepsis. The clotting cascade begins with activation of tissue factor on circulating monocytes, tissue macrophages and possibly subsets of endothelial cells, which triggers the extrinsic pathway of the coagulation system and generates thrombosis. In a randomized, double-blind, placebo-controlled trial, tissue factor expression on monocytes was markedly reduced by unfractionated heparin (UFH). Heparin blunts endotoxin-induced coagulation activation, including upstream and downstream of thrombin [28]. Although immunothrombosis plays a beneficial role in early host defense against bacterial dissemination, aberrant or uncontrolled immunothrombosis may be detrimental to the host [29]. Choosing the appropriate timing of anticoagulation is crucial; unfortunately, few studies have been designed on the basis of anticoagulation opportunities in recent decades. Our study showed that anticoagulation by heparin within 48 h after admission to the ICU was beneficial for reducing the in-hospital mortality of septic patients.

Many controversies with regard to the doses of heparin used in sepsis remain [30]. Low doses of heparin, two or three times a day, are beneficial for the prognosis of patients with sepsis [11]. An open-label, multicenter, randomized, active control trial investigating full-dose heparin vs*.* prophylactic-dose heparin in high-risk patients with sepsis induced by COVID-19 is underway [31]. In the current study, however, only administration of ≥5 heparin doses per 48 h was associated with improved in-hospital mortality in patients with sepsis. Some side effects of heparin cannot be evaluated, such as bleeding or heparin-associated thrombocytopenia (HIT), especially when used at higher doses. If the patient is deemed to be at intermediate or high risk for HIT, heparin should be stopped; if administered, its effects must be reversed by using vitamin K [32]. Thus, it is our belief that during the early stage of microcirculation disorders, appropriate heparin administration might reduce the formation of microthrombosis. In contrast, in the late stage, the use of clotting factors would lead to bleeding and other complications.

The underlying pathogenesis of sepsis appears to be complex and depends on the etiologic microorganism, site of infection and host responses. Liu *et al*. reported that unfractionated heparin alleviated sepsis-induced acute lung injury by protecting tight junctions [33]. Inhibition of caspase-11 signaling is believed to be a promising strategy for treating gram-negative bacterial sepsis [34].Tang *et al*.’s study indicated a novel role of heparin in inhibiting the caspase-11 pathway to prevent septic coagulation and lethality [35]. Animal and clinical studies have indicated that UFH modulates lipopolysaccharide-induced cytokine production by different signaling pathways [36,37]. However, the efficacy of heparin in gram-positive bacterial infection has not been reported. Our findings indicated that heparin could improve the prognosis of patients with sepsis caused not only by gram-negative bacteria but also by gram-positive bacteria. However, the underlying mechanism of heparin on gram-positive bacteria may be associated with the dormant endotoxin in our gastrointestinal tract produced every day by our trillions of bacteria in the colon, which activate by gram positive.

A dysregulated inflammatory response caused by infection may result in coagulation activation [1]. Heparin is a glycosaminoglycan that contributes to a better prognosis through its anticoagulant properties and anti-inflammatory effects [30]. It binds histones and prevents histone-mediated cytotoxicity *in vitro* and decreases mortality in sepsis without increasing the risk of bleeding [38]. Nonanticoagulant heparin attenuates histone-induced inflammatory responses in whole blood [39]. Administration of nonanticoagulant heparin is a novel and promising approach that may be further developed to treat patients suffering from sepsis. It was realized that ~70% of the heparin molecules in UFH did not bind to antithrombin (AT), a fraction which was termed ‘inactive heparin’ or ‘low-affinity material’. The ‘inactive’ heparin molecules may have contributed positively to the overall therapeutic effects of UFH [40].

Coagulopathy with sepsis or other variables, including the type of infectious source, may influence the efficacy of heparin therapy for sepsis [41]. Thus, we analyzed patients in the septic subgroup, and the results revealed that heparin improved only patients with SIC or septic shock. Moreover, heparin was effective only for septic patients with SOFA scores ≥ 10. Studies have shown that heparin alleviates the sepsis-induced renal inflammatory response and improves kidney and lung function [33,42]. It is likely that heparin is beneficial to septic patients with disseminated intravascular coagulation [43], mechanical ventilation use and shock [44]. Our study also showed that heparin reduced in-hospital mortality in patients with sepsis complicated by AKI but did not reduce the incidence of AKI in the setting of sepsis.

Of note, there are some limitations to this study. First, as this is a retrospective study, there might be measurement bias due to the long time span, although PSM analysis was used to reduce the selection bias. Second, some variables of the patients were not extracted from the database, leading to some confounding or bias. We used E-value sensitivity analysis to quantify the potential implications of unextracted confounders and found that an unextracted confounder was unlikely to explain the entirety of the treatment effect. Third, inflammatory parameters, including levels of C-reactive protein, interleukin-1β and tumor necrosis factor, were missing in the database, which made it impossible to compare the anti-inflammatory effects of heparin administration before and after use. In the same period of study, the probability of loss to follow-up was equal, and the inflammatory parameters should not have biased the HRs. Last, side effects of heparin, such as bleeding or HIT, could increase mortality in the higher dose group, resulting in the potential for the HRs in our study to be slightly underestimated.

## Conclusions

This cohort study suggested that early prophylactic heparin prescriptions may be associated with reduced risk-adjusted in-hospital mortality and 60-day mortality among septic patients, especially those with SIC, septic shock, AKI a SOFA score ≥ 10 and administration of 5 doses of heparin.

## Abbreviations

AKI: Acute kidney injury; CCI: Charlson comorbidity index; CI: Confidence interval; CKD: Chronic kidney disease; HIT: Heparin-associated thrombocytopenia; HR: Hazard ratio; ICU: Intensive care unit; MIMIC: Medical Information Mart for Intensive Care; PSM: Propensity score matching; RRT: Renal replacement therapy; SAPS II: Simplified acute physiology score II; SD: Standard deviation; SIC: Sepsis-induced coagulation; SMD: Standardized mean difference; SOFA: Sequential organ failure assessment; UFH: Unfractionated heparin.

## Authors’ contributions

All authors had full access to all the data in the study and take responsibility for the integrity of the data and the accuracy of the data analysis. YMY, MW and ZYZ were responsible for the study concept and design. ZYZ and JJH were responsible for collecting the data. ZYZ was responsible for statistical analysis. ZJY, ZPZ and JJZ were responsible for literature retrieval. MW, YYL and ZYZ were responsible for drafting the manuscript. YMY was responsible for critical reading of a final version of the manuscript.

## Funding

This work was supported by grants from the Sanming Project of Medicine in Shenzhen (SZSM20162011), The Project of Shenzhen Science and Technology Innovation Commission (JCYJ20190806163603504) and Shenzhen Second People’s Hospital Clinical Research Fund of Guangdong Province High-level Hospital Construction Project (20173357201815, 20193357003, 20203357014).

## Data availability

The datasets used in the present study are available from the first author and corresponding authors on reasonable request.

## Ethics approval and consent to participate

This study was approved by the Research Ethics Committee of Shenzhen Second People’s Hospital (20210831001). Considering the retrospective study design and depersonalization of the data, the Ethics Committee agreed to waive the requirement for written informed consent.

## Conflict of interest

None declared.

## Supplementary Material

Figure_S1_1_tkac029
