# Supplementary figures and images for: Early prophylactic anticoagulation with heparin alleviates mortality in critically ill patients with sepsis: a retrospective analysis from the MIMIC-IV database
Source: Burns Trauma. 2022 Sep 23;10:tkac029. doi: 10.1093/burnst/tkac029 (PMC9501718; doi:10.1093/burnst/tkac029)

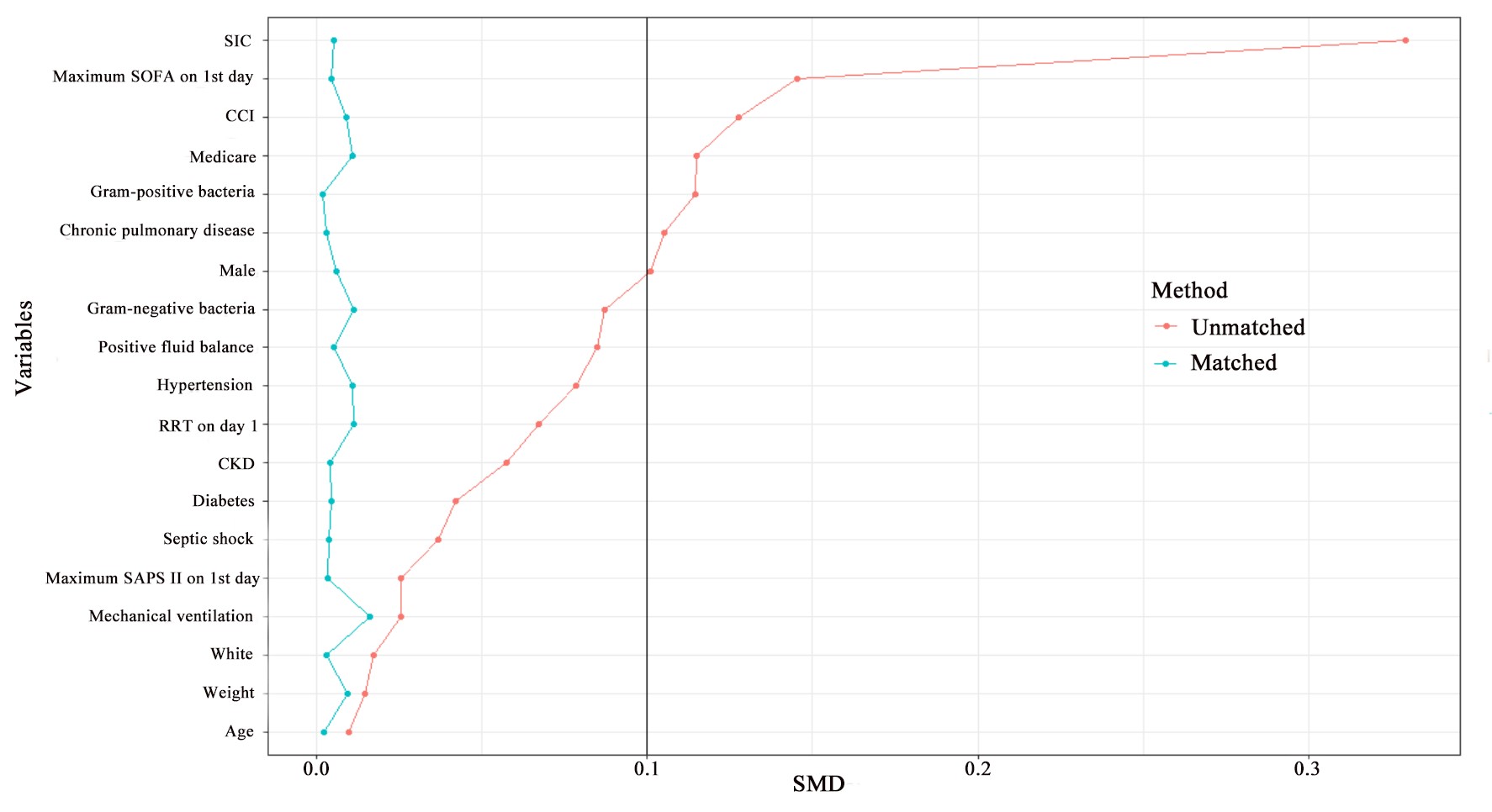

Supplement: Figure_S1_1_tkac029 [file figure_s1_1_tkac029.jpeg]
